# A straightforward method using the sign of the piezoelectric coefficient to identify the ferroelectric switching mechanism

**DOI:** 10.1038/s41598-023-34923-0

**Published:** 2023-05-31

**Authors:** Shoji Ishibashi, Reiji Kumai, Sachio Horiuchi

**Affiliations:** 1 Research Center for Computational Design of Advanced Functional Materials (CD-FMat), National Institute of Advanced Industrial Science and Technology (AIST), Tsukuba, Ibaraki 305-8568 Japan; 2 Photon Factory, Institute of Materials Structure Science, High Energy Accelerator Research Organization (KEK), Tsukuba, Ibaraki 305-0801 Japan; 3 Research Institute for Advanced Electronics and Photonics (RIAEP), National Institute of Advanced Industrial Science and Technology (AIST), Tsukuba, Ibaraki 305-8565 Japan

**Keywords:** Condensed-matter physics, Theory and computation

## Abstract

Some organic ferroelectrics have two possible switching modes: molecular reorientation and proton transfer. Typical examples include 2,5-dihydroxybenzoic acid (DHBA) and Hdabco-ReO$_4$ (dabco = diazabicyclo[2.2.2]octane). The direction and amplitude of the expected polarization depends on the switching mode. Herein a straightforward method to identify the ferroelectric switching mechanism is demonstrated. First, the relationship between the polarization vectors corresponding to the two modes is illustrated using the Berry phase. Second, the theoretical background for the sign of the piezoelectric coefficient is used to decide which mode occurs. Finally, comparing the theoretically calculated piezoelectric coefficients to the experimental results confirms the switching mode of each compound.

## Introduction

Organic ferroelectrics show practical advantages such as environmental friendliness, lightness, and flexibility^1^. Variations in the molecular degrees of freedom can divide the ferroelectric switching mechanism into roughly four types: (a) displacement, (b) reorientation, (c) proton transfer, and (d) electron transfer^2^. Recently, two possible switching mechanisms have been noted for the 2,5-dihydroxybenzoic acid (DHBA) crystal^3^. One is a flip-flop (FF) motion of hydroxy groups (mode (b)), and the other is inter-molecular proton transfer (PT) (mode (c)). Interestingly, theoretical calculations imply that the direction and amplitude of the resultant polarization vector depend on the switching mechanism^3^. This fact is reminiscent of the Berry-phase theory, which indicates that both knowledge of the crystal structure and the switching mechanism are necessary to evaluate the electric polarization. A comparison of the experimental and calculated polarization amplitudes has demonstrated that the FF mechanism is plausible in the DHBA crystal. The observed small deuteration effect on the ferroelectric properties indirectly supports this conclusion. Similarly, Hdabco-ReO$_4$ (dabco = diazabicyclo[2.2.2]octane) may show two potential switching mechanisms. Not only the relative displacements of cations and anions but also the PT process is likely responsible for the large switchable polarization^4^. Considering the nearly globular molecular shape, a different possible transition path can be imagined: rotation of Hdabco$^+$ molecules. Several organic perovskites such as Mdabco-NH$_4$-I$_3$ (Mdabco = *N*-methyl-$N'$-diazabicyclo[2.2.2]octonium) show ferroelectric switching via molecular rotation^5^.

Here, we comprehensively illustrate the relationship between the two kinds of switching modes in terms of the Berry phase for DHBA and Hdabco-ReO$_4$. (See Supplemental Material Fig. S1 for their chemical and crystal structures.) That is, the origins of the differences in the direction and amplitude of the resultant polarization vector are evaluated. Then we show that the sign of the piezoelectric coefficient can be used to identify the switching mode and to calculate piezoelectric coefficients for these two compounds. Comparing the calculated coefficients with the experimentally obtained values explicitly confirms the switching mode for each compound.

**Figure 1:**
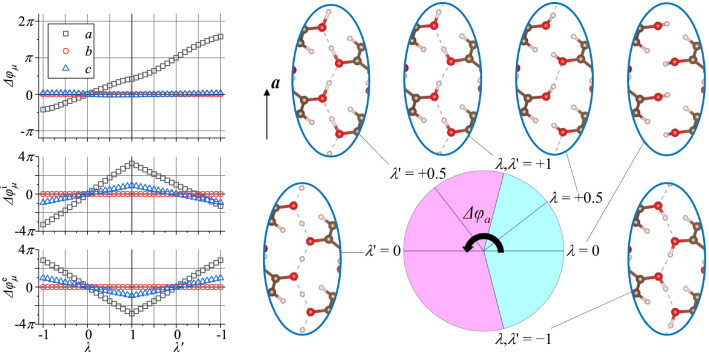
Berry phase variation $\Delta \varphi _\mu$ ($\mu = a, b, c$) as a function of $\lambda$ or $\lambda$’ for DHBA. Since there are relationships of $\Delta \varphi _a (\lambda '=-1) - \Delta \varphi _a (\lambda =-1) = 2\pi$ and $\Delta \varphi _a (\lambda '=0) - \Delta \varphi _a (\lambda =0) = \pi$, $\Delta \varphi _a$ is plotted with a circular chart to visualize the interrelationship between the $\lambda$ and $\lambda '$ processes. Insets are snapshots of the hydrogen-bonded network.

## Computational methods

The calculations in this study use our in-house QMAS code^6^, which is based on the projector augmented-wave method^7^ and a plane-wave basis set. QMAS has been utilized in various studies^8^. The PAW-potential sets were constructed according to the recipe in Ref.^9^. The Re5p5d6s, O2s2p, N2s2p, C2s2p, and H1s orbitals are treated as valence orbitals. The target ferroelectric structures are constructed from the experimental structures^3,4^ with the hydrogen-atom positions computationally optimized using the Perdew–Burke–Ernzerhof (PBE) version of the generalized gradient approximation (GGA)^10^ for the electronic exchange-correlation energy. The polarization changes are obtained via a Berry-phase formalism^11,12^. This approach has been applied to predict the spontaneous polarization for various organic ferroelectrics. The obtained results agree well with the optimized experimental values^13–17^.

To calculate direct and converse piezoelectric coefficients, since a full structural optimization of the lattice parameters and the atomic positions is necessary, we employ the van der Waals density-functional consistent-exchange (cx) method^18^ and the revised Vydrov–van Voorhis (rVV10) method^19,20^.

For reference, neither PBE nor its revised version PBEsol^21^ could reproduce the lattice parameters of DHBA and Hdabco-ReO$_4$ successfully, since they do not include the van der Waals interaction (Supplemental Material Table S1).

The cx and rVV10 functionals are implemented according to the Wu–Gygi algorithm^22,23^ based on the efficient algorithm proposed by Román-Pérez and Soler^24^. In the converse-piezoelectric-coefficient calculations, a static electric field is computationally applied according to the method proposed by Souza et al.^25^. The atomic positions were optimized using the Broyden–Fletcher–Goldfarb–Shanno (BFGS) algorithm^26^ or the fast inertial relaxation engine (FIRE) algorithm^27^ depending on the situation. The FIRE algorithm can also be used to calculate the lattice vectors. The finite basis set correction^28^ is applied to evaluate the stress components. Our previous studies successfully utilized these computational techniques^29,30^. In this study, the plane-wave cutoff energy is set to 20 Ha. The number of ${\varvec{k}}$ points in the full Brillouin zone is $12 \times 6 \times 6$ and $6 \times 6 \times 8$ for DHBA and Hdabco-ReO$_4$, respectively. The convergence criteria are $5 \times 10^{-5}$ Ha/bohr for the maximum force and $2 \times 10^{-7}$ Ha/bohr$^3$ for the square root of the sum of the squares of the stress components.

## Experimental methods

The Berlincourt method^31^ is used to measure the direct piezoelectric coefficient. The converse piezoelectric coefficient is determined by measuring the longitudinal strain, which is synchronized with the *P*–*E* hysteresis experiments. Further details are described in Ref.^32^.

To validate the computationally obtained lattice parameters, the 0-K extrapolated lattice parameters are obtained by linear regression analyses of the experimental results at several temperatures where the room-temperature structures are kept. Data taken at 127, 145, and 162 K (DHBA) and 200, 223, and 240 K (Hdabco-ReO$_4$) are used in the analyses. (See Supplemental Material Figs. S2–S3 for the experimental details of the corresponding diffraction studies using synchrotron radiation.)

## Results and discussion

The FF and PT switching modes in DHBA occur at the 5-hydroxy groups, forming a zigzag OH$\cdots$O hydrogen-bonded sequence along ${\varvec{a}}$. For the FF process, which is identified as the actual mode in the previous study^3^, we introduce a parameter $\lambda$ and describe the target ferroelectric structure as $\lambda =+1$. The polarization-inverted structure is described as $\lambda =-1$. From $\lambda =+1$ to $-1$, a switching hydrogen atom rotates around the adjacent C–O bond by $\theta \sim$ 162$^{\circ }$. Structural models at intermediate $\lambda$ values ${\varvec{S}}(\lambda )$ are constructed as $(1/2+\lambda /2) {\varvec{S}}_+ + (1/2-\lambda /2) {\varvec{S}}_-$, where ${\varvec{S}}_+$ is the structure prepared from ${\varvec{S}}(\lambda =+1)$ with the switching hydrogen atoms rotated by $(1/2-\lambda /2)\theta$ while ${\varvec{S}}_-$ is that prepared from ${\varvec{S}}(\lambda =-1)$ with the rotations by $-(1/2+\lambda /2)\theta$. The structure at $\lambda =0$ is centrosymmetric. Similarly, as for the PT mode, we introduce a parameter $\lambda '$. The target ferroelectric structure with $\lambda '=+1$ is the same as that for $\lambda =+1$ and its inversion is described as $\lambda '=-1$. Crystallographically, the structure for $\lambda '=-1$ is equivalent to that for $\lambda =-1$. Structural models at intermediate $\lambda '$ values ${\varvec{S}} (\lambda ')$ are constructed as $(1/2+\lambda '/2){\varvec{S}}$ $(\lambda '=+1) + (1/2-\lambda '/2) {\varvec{S}}(\lambda '=-1)$. Although not a requirement, if $\lambda =\lambda '$, the atomic positions, except for the four switching hydrogens, are the same.

**Figure 2:**
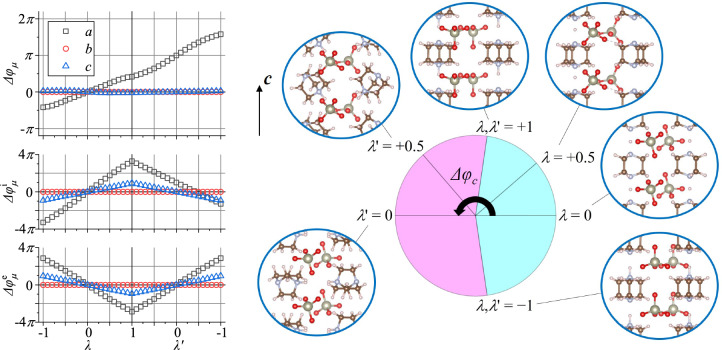
Berry phase variation $\Delta \varphi _\mu$ ($\mu = a, b, c$) as a function of $\lambda$ or $\lambda$’ for Hdabco-ReO$_4$. Similar to Fig. 1, $\Delta \varphi _c$ is plotted with a circular chart. Insets represent structural snapshots at several $\lambda$ or $\lambda '$ values.

The relationship between the two modes can be clearly understood using the Berry-phase description. The total polarization change $\Delta {\varvec{P}}$ is obtained as the sum of the ionic contribution $\Delta {\varvec{P}}^{\text{i}}$ and the electronic contribution $\Delta {\varvec{P}}^{\text{e}}$. Here, the $\lambda =0$ state is set to the origin of the polarization. For a spin-degenerate case, the total Berry-phase change along a particular crystallographic direction $\mu$ ($= a$, *b*, or *c*) is represented as

1 $$\begin{aligned} \Delta \varphi _\mu = \frac{\Omega }{2|e |} {\varvec{G}}_\mu \cdot \Delta {\varvec{P}}, \end{aligned}$$

where ${\varvec{G}}_\mu$ is the reciprocal lattice vector corresponding to $\mu$. Similarly, the ionic and electronic parts are described by

2 $$\begin{aligned} \Delta \varphi _\mu ^{\text{i}} = \frac{\Omega }{2|e |} {\varvec{G}}_\mu \cdot \Delta {\varvec{P}}^{\text{i}}, \ \ \ \ \ \Delta \varphi _\mu ^{\text{e}} = \frac{\Omega }{2|e |} {\varvec{G}}_\mu \cdot \Delta {\varvec{P}}^{\text{e}}. \end{aligned}$$

We evaluate $\Delta \varphi _\mu$ from $\lambda =-1$ to $\lambda '=-1$ via $\lambda =0$, $\lambda =\lambda '=+1$, and $\lambda '=0$. Again, the $\lambda =+1$ state is the same as the $\lambda '=+1$ state. Figure 1 plots the results. As a matter of course,

3 $$\begin{aligned}{} & {} {\Delta \varphi _\mu (\lambda =+1) - \Delta \varphi _\mu (\lambda =0)} \nonumber \\{} & {} \quad = -(\Delta \varphi _\mu (\lambda =-1) - \Delta \varphi _\mu (\lambda =0)), \end{aligned}$$

4 $$\begin{aligned}{} & {} {\Delta \varphi _\mu (\lambda '=+1) - \Delta \varphi _\mu (\lambda '=0)} \nonumber \\{} & {} \quad = -(\Delta \varphi _\mu (\lambda '=-1) - \Delta \varphi _\mu (\lambda '=0)). \end{aligned}$$

Two key features observed for $\mu =a$: $\Delta \varphi _a (\lambda '=-1) - \Delta \varphi _a (\lambda =-1) = 2\pi$ and $\Delta \varphi _a (\lambda '=0) - \Delta \varphi _a (\lambda =0) = \pi$.

These relations can be visualized as a circular chart along with structure snapshots (Fig. 1). Comparing the $\Delta \varphi _a$ plot with those of $\Delta \varphi _a^{\text{i}}$ and $\Delta \varphi _a^{\text{e}}$ shows that the $2\pi$ difference between $\lambda =-1$ and $\lambda '=-1$ and the $\pi$ difference between $\lambda =0$ and $\lambda '=0$ are due to the ionic contribution. The crystal structures at $\lambda =-1$ and $\lambda '=-1$ are crystallographically equivalent, whereas the fractional coordinates of the four switching hydrogen atoms change by $(0.480, -0.027, 0.016)$, $(0.520, 0.027, -0.016)$, (0.480, 0.027, 0.016), and $(0.520, -0.027, -0.016)$, respectively. Their sum of (2, 0, 0) is consistent with the polarization change $2|e |{\varvec{R}}_a/\Omega$, which corresponds to the Berry phase change of $2\pi$ (because ${\varvec{G}}_a \cdot {\varvec{R}}_a = 2\pi )$. As for the difference between $\lambda =0$ and $\lambda '=0$, the changes in the fractional coordinates of the four hydrogens are $(0.218, -0.044, -0.053)$, (0.282, 0.044, 0.053), $(0.218, 0.044, -0.053)$, and $(0.282, -0.044, 0.053)$, respectively. Their sum of (1, 0, 0) is consistent with the $\pi$ difference in the Berry phase. In principle, the Berry-phase difference between two centrosymmetric structures should be $n\pi$, where *n* is an integer.

The spontaneous polarization vector for the FF process can be reconstructed via

5 $$\begin{aligned} \frac{1}{2\pi }\frac{2|e |}{\Omega }\sum _\mu (\Delta \varphi _\mu (\lambda =+1) - \Delta \varphi _\mu (\lambda =0)) {\varvec{R}}_\mu , \end{aligned}$$

where ${\varvec{R}}_\mu$ is the lattice vector corresponding to ${\varvec{G}}_\mu$. Setting Cartesian coordinates *x*, *y*, and *z* parallel to ${\varvec{R}}_a$, ${\varvec{R}}_b$, and ${\varvec{G}}_c$, the polarization vector is represented as $(5.05, 0, -0.69)$ $\upmu$C/cm$^2$. Similarly, the polarization vector for the PT process can be obtained from $\Delta \varphi _\mu (\lambda '=+1)$ and $\Delta \varphi _\mu (\lambda '=0)$. The result is $(-6.98, 0, -0.69)$ $\upmu$C/cm$^2$. These values are very similar to those reported previously^3^. The sign of the *x*-component differs between the FF and PT processes.

Similar analyses were performed for Hdabco-ReO$_4$. The Hdabco$^+$ molecules construct a linear NH$\cdots$N hydrogen-bonded sequence along ${\varvec{c}}$. In this case, $\lambda$ is assigned to the PT process, which is thought to actually occur while $\lambda '$ is assigned to the FF process. Both switching processes are slightly more complicated than those in the DHBA case as only the hydrogen atoms at the hydroxy groups move significantly in the DHBA case. The PT process has an inter-molecular hydrogen transfer between Hdabco$^+$ molecules, whereas the Hdabco$^+$ molecules are rotated by 180$^{\circ }$ in the FF process. In addition, both processes show small relative displacements of ions and reorientation of ReO$_4^-$ tetrahedra. Figure 2 plots the variation of the Berry phase as a function of $\lambda$ or $\lambda '$ via a circular chart and structure snapshots. The relationships among the $\Delta \varphi _\mu$ values at $\lambda ,\lambda ' = 0, \pm 1$ in the Hdabco-ReO$_4$ case are the same as those in the DHBA case (Eqs. 3 and 4). The behavior of $\Delta \varphi _c$ is similar to that of $\Delta \varphi _a$ for DHBA. The phase change is along the hydrogen-bond direction in each compound. For Hdabco-ReO$_4$, $\Delta \varphi _c (\lambda '=-1) - \Delta \varphi _c (\lambda =-1) = 2\pi$ and $\Delta \varphi _c (\lambda '=0) - \Delta \varphi _c (\lambda =0) = \pi$. Again, the $2\pi$ and $\pi$ differences reflect the positions of two NH hydrogen atoms in the unit cell. From $\lambda =-1$ to $\lambda '=-1$, each hydrogen atom travels the unit-cell length along ${\varvec{c}}$.

**Table 1:** Lattice parameters of DHBA and Hdabco-ReO$_4$. The “EXP0” column lists the 0-K extrapolated values obtained by linear regression analyses on the X-ray diffraction results. Values in parenthesis represent deviations from the EXP0 values (as a percentage).

	DHBA			Hdabco-ReO$_4$		
	EXP0	cx	rVV10	EXP0	cx	rVV10
*a* (Å)	4.8719	4.7990 (−1.5)	4.8414 (−0.6)	10.049	10.279 ($+$2.3)	10.008 (−0.4)
*b* (Å)	11.801	11.974 ($+$1.5)	11.859 ($+$0.5)	8.685	9.036 ($+$4.0)	8.827 ($+$1.6)
*c* (Å)	10.910	11.339 ($+$3.9)	10.809 (−0.9)	5.3108	5.3013 (−0.2)	5.3325 ($+$0.4)
$\beta (^{\circ }$)	91.514	91.966	91.702	89.94	89.801	90.051

The polarization vector for Hdabco-ReO$_4$ is evaluated as $(-5.78, 0, 7.92)$ $\upmu$C/cm$^2$ in the PT mode, whereas that in the FF mode is $(-5.52, 0, -9.53)$ $\upmu$C/cm$^2$. The sign of the *z*-component (*x*-component) for Hdabco-ReO$_4$ (DHBA) differs between the two modes. The *z*-direction is parallel to the $c^*$-direction and is nearly parallel to the hydrogen-bonded network along ${\varvec{c}}$. The relatively large *x*-component for Hdabco-ReO$_4$ is attributed to the relative displacements of cations and anions. It should be noted that the amplitude of the previous experimental value for the *z*-component obtained by the pyroelectric-charge measurement (16.5 $\upmu$C/cm$^2$)^4^ is significantly larger than the present computational results regardless of the switching mode. However, the experimental *x*-component amplitude is in a similar range as the present results. Currently, the origin of the discrepancy in the *z*-component amplitude is unclear. By contrast, the value of 8.5 $\upmu$C/cm$^2$ obtained in the present study (Supplemental Material Fig. S4) agrees well with the aforementioned calculated value for the PT mode.

Next, we explain why the sign of the piezoelectric coefficient can identify the switching mode. If stress is applied along a direction where finite polarization is observed, the polarization changes from ${\varvec{P}}$ to ${\varvec{P}}+\Delta {\varvec{P}}$. This is the direct piezoelectric effect. Since the piezoelectric effect is due to a small deformation and is not accompanied by the ferroelectric switching, the change in polarization as a vector $\Delta {\varvec{P}}$ is uniquely defined and independent of the ferroelectric switching mechanism. Generally, $|{\varvec{P}} |\gg |\Delta {\varvec{P}} |$. Here, for simplicity, we assume that only the *x*-components have finite values of $P_x$ and $P_x + \Delta P_x$ and that $\Delta P_x$ is positive. Again, $|P_x |\gg |\Delta P_x |$. If $P_x$ is positive, $|P_x |< |P_x + \Delta P_x |$, whereas if $P_x$ is negative, $|P_x |> |P_x + \Delta P_x |$. The direct piezoelectric coefficient can be evaluated from the change in the polarization amplitude upon applying stress. Thus, the sign of the direct piezoelectric coefficient depends on the sign of $P_x$. For both DHBA and Hdabco-ReO$_4$, because the signs of the polarization components along the hydrogen-bond directions differ according to the switching mechanism, the sign of the direct piezoelectric coefficient can elucidate the actual switching process.

The converse piezoelectric effect corresponds to the strain as a first-order response against an induced electric field ${\varvec{E}}$. We assume that the polarization and electric-field vectors have finite amplitudes only for their *x*-components $P_x$ and $E_x$. By applying $E_x$, a finite strain ${\varvec{\varepsilon }}$ is induced. Here $\varepsilon _{xx}$ is assumed to be positive. Generally, the converse piezoelectric coefficient is evaluated by adjusting the $E_x$ sign to be the same as that of $P_x$. If the sign of $P_x$ differs, the sign of the evaluated converse piezoelectric coefficient should differ. Hence, the switching modes can be distinguished.

To evaluate the piezoelectric coefficients, crystal structures under a finite stress or electric field must be predicted accurately. Initially, we confirmed that the lattice parameters of DHBA and Hdabco-ReO$_4$ are well reproduced by the cx and rVV10 methods. Table 1 lists the obtained and 0-K extrapolated values. The calculated lattice parameters agree well with the experimental 0-K extrapolated lattice parameters.

**Table 2:** Polarization vectors ($\upmu$C/cm$^2$) of DHBA evaluated by cx or rVV10 assuming the FF or PT process.

	FF	PT
cx	$(5.62, 0., -0.48)$	$(-6.42, 0., -0.48)$
rVV10	$(5.77, 0., -0.49)$	$(-6.26, 0., -0.49)$

**Figure 3:**
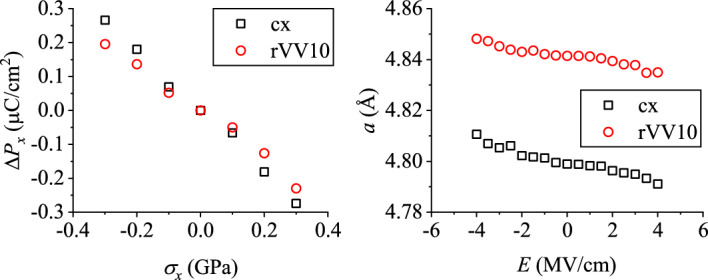
Simulated direct and converse piezoelectric effects for DHBA.

The polarization vectors were evaluated on these computationally optimized structures. Table 2 lists the results for DHBA. Except for a small numerical difference, the overall features are similar to those for the aforementioned GGA results for the experimental structure with the hydrogen positions computationally optimized. The *x*-component of polarization $P_x$ is positive for the FF mode, but negative for the PT mode. As a function of uniaxial stress $\sigma _x$ along ${\varvec{a}}$, its changes $\Delta P_x$ were calculated (Fig. 3, left). Here, the tensile stress corresponds to a positive value of $\sigma _x$. As previously mentioned, $\Delta P_x$ is independent of the switching mode. As $\sigma _x$ increases, $|P_x+\Delta P_x |$ decreases for the FF mode but it increases for the PT mode. We evaluated the direct piezoelectric coefficient $d_{11}$ as

6 $$\begin{aligned} d_{11} = \frac{1}{2\pi }\frac{2|e |}{\Omega }\sum _\mu \frac{\text{d} \varphi _\mu }{\text{d} \sigma _1} \varvec{R}_{\mu 1}. \end{aligned}$$

For the FF (PT) mode, the resultant $d_{11}$ value is $-8.86$ ($+8.86$) pC/N by cx or $-6.79$ ($+6.79$) pC/N by rVV10. The experimental $d_{11}$ value is $-7.7$ pC/N. The sign for the FF mode is consistent with the experiment, and their amplitudes agree reasonably well.

We also simulated the converse piezoelectric effect. Figure 3 (right) shows the variation of the lattice parameter *a* as a function of the electric field ${\varvec{E}} (\parallel x)$. The sign of the converse piezoelectric coefficient is negative for the FF process. For the PT process, since the *x*-component of the polarization vector is negative, the sign of $E_x$ should be inverted and the sign of the piezoelectric coefficient becomes positive. The converse piezoelectric coefficient $d_{11}$ is $-4.17$ ($+4.17$) pm/V by cx or $-2.95$ ($+2.95$) pm/V by rVV10. The experimental $d_{11}$ value (Supplemental Material, Fig. S4) is $-4.8$ pm/V. Again, the sign for the FF mode is consistent with the experiment. From the signs of the direct and converse piezoelectric coefficients, it is concluded that the FF process is responsible for the ferroelectric switching of DHBA.

**Table 3:** Polarization vectors ($\upmu$C/cm$^2$) of Hdabco-ReO$_4$ evaluated by cx or rVV10 assuming the PT or FF modes.

	PT	FF
cx	$(-5.72, 0., 7.23)$	$(-5.46, 0., -10.22)$
rVV10	$(-5.71, 0., 8.20)$	$(-5.45, 0., -9.25)$

**Figure 4:**
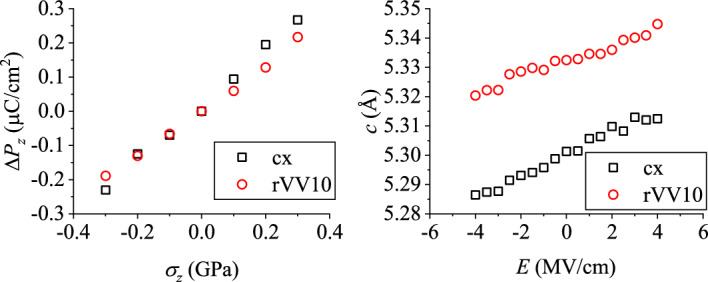
Simulated direct and converse piezoelectric effects for Hdabco-ReO$_4$.

Table 3 lists the calculated polarization vectors for Hdabco-ReO$_4$. (The order of the switching modes PT or FF are reversed from the DHBA case.) The sign of $P_z$ is positive for the PT mode, but negative for the FF mode. The *z* direction is parallel to the reciprocal vector ${\varvec{c}}^*$ and is nearly parallel to ${\varvec{c}}$ because $\beta$ is almost 90$^{\circ }$.

As for Hdabco-ReO$_4$, a stress or an electric field is applied along *z*, which is parallel to the hydrogen-bonded-network direction. Figure 4 (left) represents $\Delta P_z$ as a function of $\sigma _z$. With $\sigma _z$ increasing, $|P_z+\Delta P_z |$ increases for the PT mode while it decreases for the FF mode due to the $P_z$ sign. We evaluated the direct piezoelectric coefficient $d_{33}$ using an equation similar to Eq. (6). For the PT (FF) mode, the resultant value is $+8.20$ ($-8.20$) pC/N by cx or $+6.64$ ($-6.64$) pC/N by rVV10. Experimentally, the obtained $d_{33}$ value is $+5.1$ pC/N. The plus sign of the experimental results is consistent with the PT-mode switching.

Although the experimental results of the converse piezoelectric effect for Hdabco-ReO$_4$ are unavailable, we implemented simulations. Figure 4 (right) plots the variation of the lattice parameter *c* as a function of the electric field ${\varvec{E}} (\parallel z)$. The evaluated corresponding converse piezoelectric coefficient $d_{33}$ is $+6.86$ ($-6.86$) pm/V by cx or $+5.03$ ($-5.03$) pm/V by rVV10. The sign is consistent with that of the direct-piezoelectric-effect case.

The origin of the sign of the direct piezoelectric coefficient for each mode can be explained as follows. With a tensile stress along the hydrogen-bond direction, the hydrogen-bond length increases. Generally, the longer the hydrogen bond, the shorter the O–H or N–H bond (see, e.g., Fig. 4 in Ref.^30^). Hence, the shift of the switching hydrogen becomes smaller for the FF mode, whereas it becomes larger for the PT mode. As a result, the polarization amplitude decreases (increases) for the FF (PT) mode.

As described above, DHBA takes the FF process while Hdabco-ReO$_4$ takes the PT process. A possible reason is as follows. For DHBA, the O$\cdots$O distance $d_{{\text {O}}\cdots {\text {O}}}$ values at the switching parts are 2.73 and 2.77 Å, which are significantly longer than those for typical PT ferroelectrics with the O–H$\cdots$O hydrogen bond (CRCA, PhMDA, HPLN, and CBDC: $d_{{\text {O}}\cdots {\text {O}}}$ = 2.55–2.63 Å^16^) as already mentioned in Ref.^3^. This is an unfavorable condition for the PT process. By contrast, the $d_{{\text {N}}\cdots {\text {N}}}$ for Hdabco-ReO$_4$ is 2.86 Å and this value is close to the lower end of those for typical PT ferroelectrics with the N–H$\cdots$N hydrogen bond (MBI, DC-MBI, and ALAA: $d_{{\text {N}}\cdots {\text {N}}}$ = 2.86–2.98 Å^16^). This is a favorable condition for the PT process. In addition, in terms of the FF motion, only small parts move for DHBA while large molecules should rotate for Hdabco-ReO$_4$.

## Conclusions

The interrelationships of the two possible ferroelectric switching modes for DHBA and Hdabco-ReO$_4$ are theoretically explained in terms of the Berry phase. The piezoelectric-coefficient sign can be utilized to determine which mode actually occurs. The calculated direct and converse piezoelectric coefficients are compared with the experimental results. DHBA employs the FF process for its switching mechanism, whereas Hdabco-ReO$_4$ adopts the PT process. The present study demonstrates a straightforward method to identify the ferroelectric switching mechanism. The sign of the piezoelectric coefficient can be easily assessed even on the mesoscopic scale (e.g., using piezo force microscopy). The present study opens a novel utilization purpose of such an experimental technique. Furthermore, application of the present approach is not limited to organic materials alone.

## Supplementary Information

Supplementary Information.

## Data Availability

The datasets used and/or analyzed during the current study available from the corresponding author on reasonable request.
