# Supplementary material for: A straightforward method using the sign of the piezoelectric coefficient to identify the ferroelectric switching mechanism
Source: Sci Rep. 2023 May 31;13:8810. doi: 10.1038/s41598-023-34923-0 (PMC10232487; doi:10.1038/s41598-023-34923-0)
Supplement: Supplementary file 1 — Supplementary Information. [file 41598_2023_34923_MOESM1_ESM.pdf]

# Supplemental Material: A straightforward method using the sign of the piezoelectric coefficient to identify the ferroelectric switching mechanism

Shoji Ishibashi

*Research Center for Computational Design of Advanced Functional Materials (CD-FMat),*
*National Institute of Advanced Industrial Science and Technology (AIST),*
*Tsukuba, Ibaraki 305-8568, Ibaraki, Japan*

Reiji Kumai,

*Photon Factory, Institute of Materials Structure Science,*
*High Energy Accelerator Research Organization (KEK),*
*Tsukuba, Ibaraki 305-0801, Japan*

Sachio Horiuchi

*Research Institute for Advanced Electronics and Photonics (RIAEP),*
*National Institute of Advanced Industrial Science and Technology (AIST),*
*Tsukuba, Ibaraki 305-8565, Japan*

## Materials and Crystal Structures

Colorless rod crystals of purified DHBA were grown by sublimation under reduced pressure as described in ref [1]. Colorless rod crystals of Hdabco-$ReO_4$ were grown by slow evaporation of a 1:1 mixed aqueous solution of dabco and $HReO_4$.

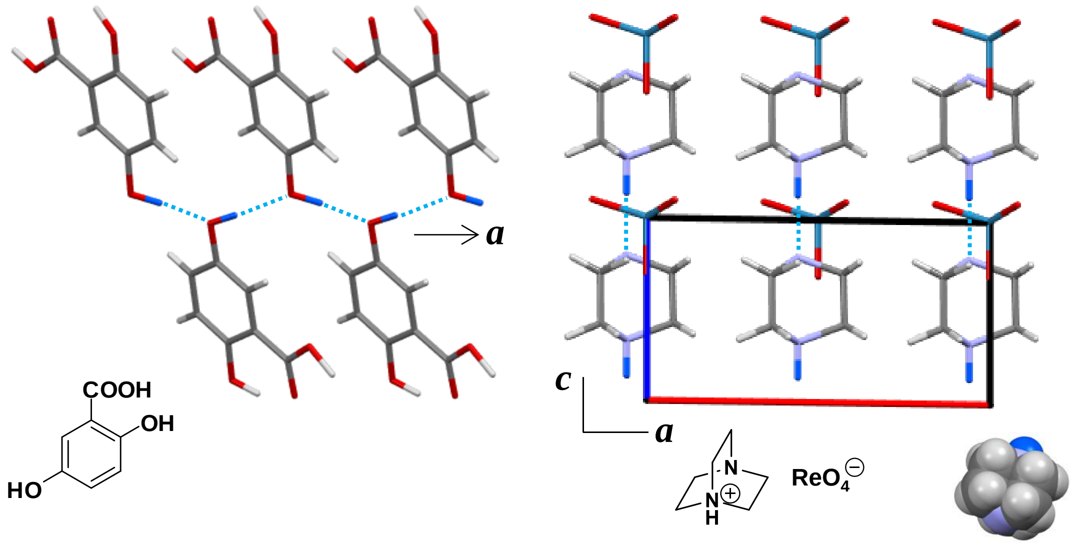


(a) DHBA (b) Hdabco-$ReO_4$

**FIG. S1.** Chemical and crystal structures of (a) DHBA and (b) Hdabco-$ReO_4$ crystals. Dotted lines indicate OH···O or NH···N hydrogen bonds. Atomic coordinates are from refs [1,2].

## Lattice parameters obtained by PBE and PBEsol

Lattice parameters of DHBA and Hdabco-$ReO_4$ were calculated using the PBE and PRBsol functionals. The results are shown in Table S1 together with the 0-K extrapolated values (EXP0) from the X-ray diffraction experimental results (See the next section). For DHBA, both the PBE and PBEsol functionals overestimate the lattice parameter *c*. Along this direction, there is not hydrogen-bond network and the van der Waals interaction is thought to play an important role for binding. As for Hdabco-$ReO_4$, because of its ionic nature, the overestimation of the lattice parameters along which no hydrogen-bond network exists is reduced but still significant. The PBEsol functional is a revised version of the PBE functional to improve equilibrium properties such as lattice parameters of densely packed solids. However, it does not include the van der Waals interaction and failed to reproduce the lattice parameters of DHBA and Hdabco-$ReO_4$.

**Table S1.** Lattice parameters of DHBA and Hdabco-$ReO_4$ obtained by PBE and PBEsol. Values in parenthesis represent deviations from the EXP0 values (as a percentage).

| | DHBA | | | Hdabco-$ReO_4$ | | |
|---|---|---|---|---|---|---|
| | EXP0 | PBE | PBEsol | EXP0 | PBE | PBEsol |
| *a* (Å) | 4.8719 | 4.9132 (+0.8) | 4.7060 (−3.4) | 10.049 | 10.542 (+4.9) | 10.425 (+3.7) |
| *b* (Å) | 11.801 | 12.021 (+1.9) | 11.889 (+0.7) | 8.685 | 9.479 (+9.1) | 9.032 (+4.0) |
| *c* (Å) | 10.910 | 12.842 (+17.7) | 12.160 (+11.5) | 5.3108 | 5.3859 (+1.4) | 5.2479 (−1.2) |
| *β* (°) | 91.514 | 86.547 | 89.144 | 89.941 | 90.147 | 88.982 |

**Temperature Dependence of the Lattice Parameters**

The temperature dependences of the lattice constants of DHBA (Fig. S2) and Hdabco-$ReO_4$ (Fig. S3) were determined by X-ray diffraction (XRD). XRD measurements were carried out using a Rigaku cylindrical imaging plate diffractometer installed in beamline BL-8B at the Photon Factory (PF), High Energy Accelerator Research Organization (KEK). The wavelength of the synchrotron X-rays was 0.6835 Å. A single crystal of DHBA or Hdabco-$ReO_4$ was fixed to a glass rod, and the temperature of the crystal was controlled by the temperature of the regulated nitrogen gas. Data correction and refinement of the lattice constants were conducted using the RAPID-AUTO software (Rigaku).

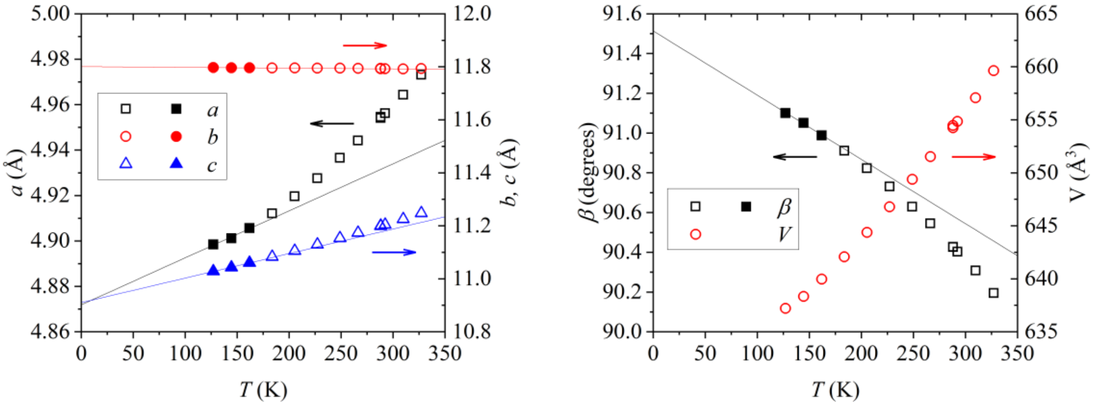


**FIG. S2.** Temperature dependence of the lattice parameters of a DHBA single crystal. Closed symbols represent data points used in the linear regression. Solid lines for *a, b, c,* and *β* represent the fitting results.

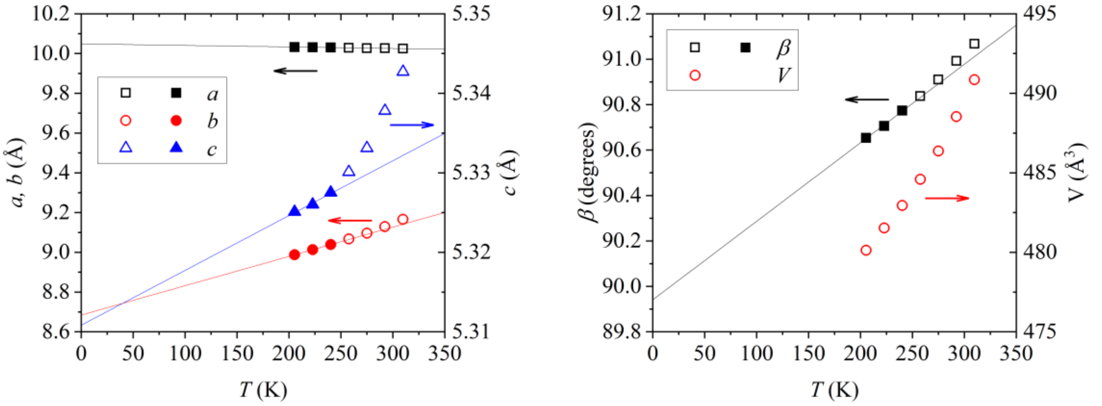


**FIG. S3.** Temperature dependence of the lattice parameters of a Hdabco-$ReO_4$ single crystal. Closed symbols represent data points used in the linear regression. Solid lines for *a, b, c,* and *β* denote the fitting results.

**Polarization Hysteresis and Piezoelectric Measurements**

The crystals were electrically poled with an external field (in the *a* and *c* direction for DHBA and Hdabco-$ReO_4$ crystals, respectively) because a fully polarized state is necessary to evaluate the intrinsic piezoelectric properties. The electric polarization and electric field (*P*−*E*) hysteresis measurements and poling at room temperature were conducted on a ferroelectrics evaluation system (FCE-1; Toyo) consisting of a current/charge−voltage converter (model 6252), an arbitrary waveform generator (Biomation 2414B), an analogue-to-digital converter (WaveBook 516), and a voltage amplifier (HVA4321; NF). The crystals were immersed in silicone oil to prevent atmospheric discharge under a high electric field. The longitudinal piezoelectric coefficient was measured using a piezometer (PM300, PiezoTest) calibrated with a PZT disk with a known $d_{33}$ under short-circuit conditions (constant $E = 0$) via the Berlincourt method. The poled specimen was clamped at its two electrode-painted crystal surfaces and a constant force of 0.3–1.0 N was applied. The piezoelectric charge resulting from the action of an additional dynamic force of 0.05–0.10 N (rms) oscillating at a frequency of 110 Hz was then measured. The longitudinal piezoelectric coefficient of the converse effect was determined by measuring the longitudinal strain, which was synchronized with the *P*−*E* hysteresis experiments by additionally equipping the FCE-1 ferroelectrics evaluation system described above with a heterodyne interferometer (LV-2100; Ono Sokki). The fully poled single crystal was completely immersed in insulating silicone oil and sandwiched between the bottom electrode and a thin aluminum-rod electrode (3-mm diameter, 4-cm long, and weighing 1.29 g with a 0.5-mm-diameter tip) was placed on it. The $d_{11}$ value of DHBA was determined from the slope of the linear strain effect at an approximately zero field (Fig. S4).

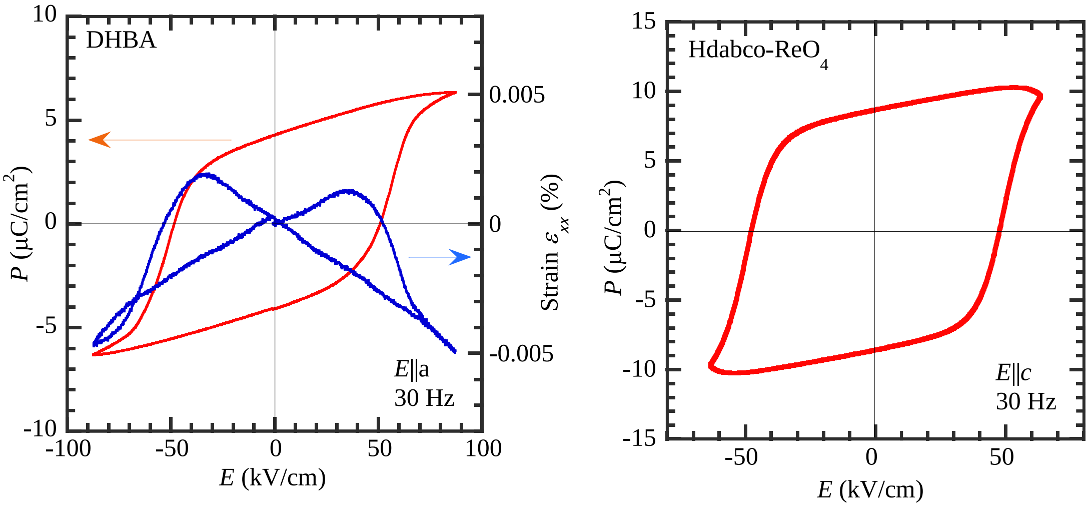


**FIG. S4.** *P*-*E* hysteresis loops of the DHBA (left) and Hdabco-$ReO_4$ crystals (right) used in poling for piezoelectric measurements. Simultaneously measured longitudinal strain (blue curve) is also shown for DHBA.

## References

[1] Y. Shimoi, S. Tsuzuki, R. Kumai, M. Sotome, and S. Horiuchi, J. Mater. Chem. C **10**, 10099 (2022).

[2] M. Szafrański, A. Katrusiak, and G. J. McIntyre, Phys. Rev. Lett. **89**, 215507 (2002).
